# An intracapsular nephrectomy for the acquired cystic disease-associated renal cell carcinoma in renal transplant allograft: A clinical case report

**DOI:** 10.1097/MD.0000000000025858

**Published:** 2021-05-14

**Authors:** Yue Song, Jingjing Zheng, Shiying Guo, Lianhui Fan

**Affiliations:** a Department of Urology; b Department of Anesthesia, General Hospital of Northern Theater Command, Shenyang, China.

**Keywords:** acquired cystic disease-associated renal cell carcinoma, nephrectomy, renal allograft

## Abstract

**Rationale:**

Acquired cystic disease-associated renal cell carcinoma (ACKD-RCC) is a unique subtype of renal cell carcinoma (RCC) and is found exclusively in patients with end-stage renal disease. We report a case of intracapsular nephrectomy (ICAN) of renal allograft with ACKD-RCC. To our knowledge, this is the first case in Asia of ICAN of renal allograft to treat ACKD-RCC.

**Patient concerns:**

A 51-year-old male patient with a history of allogeneic kidney transplantation (23 years previously) presented with renal cystic degeneration of the transplanted kidney over the past 2 years.

**Diagnoses:**

ICAN was used to remove the cystic kidney.

**Interventions:**

The pathology report indicated clear cell renal cell carcinoma.

**Outcomes:**

Two years after surgery, computed tomography showed no tumor recurrence, and the patient's creatinine level was 3.5 mg/dl under hemodialysis.

**Lessons:**

Removal of transplanted kidney with ACKD-RCC using ICAN is feasible to provide a mid-term tumor-free survival for the patient. Therefore, we consider nephrectomy as an early treatment for the nonfunctional cystic allograft kidney, in order to reduce the dosage of anti-rejection drugs, avoid the occurrence of transplanted kidney tumor, and provide the possibility for the patient an opportunity to receive a second kidney transplantation.

## 1 Introduction

Acquired cystic disease-associated renal cell carcinoma (ACKD-RCC), described originally by Tickoo et al, is found exclusively in end-stage renal disease (ESRD) well before receiving dialysis, and is not common in transplanted kidneys suffering chronic rejection.^[1–3]^ ACKD-RCC was first officially recognized as a distinct RCC entity in 2013 by the International Society of Urological Pathology. However, there is limited data on the genetic aberrations of this tumor, and the pathogenesis of this neoplasm is also uncertain. It is reported that ACKD-RCC has relatively more aggressive behavior than other RCC subtypes seen in the setting of ESRD.^[3]^ There are currently no guidelines on the management of de novo graft tumors in renal transplant patients. Here we described a case of intracapsular nephrectomy (ICAN) of renal allograft with ACKD-RCC.

## 2 Case report

**Figure 1:**
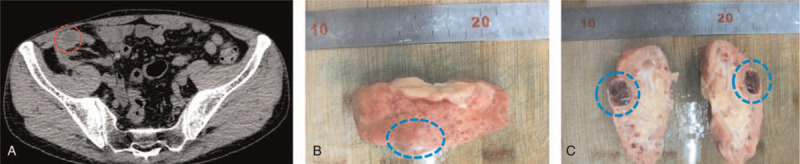
Gross specimen observation of transplanted kidney. A. A CT scan shows a convex growth tumor on the ventral cortex of the atrophy renal transplant. The tumor has smooth edges, uneven internal CT values, and a mixed density of cysts and solid tissue. B. The gross specimen shows a convex tumor in the cortex of the renal transplant. C. After longitudinal incision, the tumor capsule is intact, and its inside appears cystic degeneration, with focal hemorrhage and necrosis.

A 51-year-old male was diagnosed with glomerulonephritis by renal biopsy 25 years ago, and developed into chronic renal failure (uremia period), then received allogeneic kidney transplantation 2 years later. Cyclosporine A, azathioprine and a small dose of prednisolone were used to inhibit the immune response and reduce the rejection. The serum creatinine was lower than 2.3 mg/dL in the first 10 years after transplantation. Renal insufficiency began 15 years after transplantation. Other accompanying symptoms gradually appeared, including increased serum creatinine level, decreased urine volume, facial and lower extremities edema, hypertension, proteinuria, etc. There was little improvement of renal function after methylprednisolone shock therapy and renal nutrition treatment. The patient's condition worsened to uremia 15.5 years after transplantation and began regular hemodialysis. 21 years after transplantation, CT scan showed that there was a convex growth cyst-solid mixed mass in the cortex of the transplanted kidney. The volume was about 1.5 cm × 1.0 cm × 0.5 cm (Fig. 1A), which was increased to 2.5 cm × 1.5 cm × 1.5 cm in the 22nd year after transplantation. Considering that the transplanted kidney had lost its function and ruling out the possibility of tumor metastasis to surrounding tissues and distant organs, the patient received ICAN under general anesthesia.

The brief procedures of the operation were as follows. First, we exposed the transplanted kidney outside the peritoneum through an incision at the outer edge of the right lower rectus abdominis. We observed that the kidney adhered tightly to the surrounding tissues and could not be separated completely. Considering that sharp separation or forced dissection may cause serious damage to the surrounding organs or renal pedicle vessels, we decided to use ICAN. The inflammatory adhesive renal capsule was cut longitudinally along the long axis of the kidney, and separated with a vascular clamp in order to find out the space between the renal capsule and the cortex. The index finger was then extended into the space, and blunt separation was performed in the anterior, posterior, upper, and lower directions of the involved kidney. The adhesion of the renal pedicle at the hilum of the kidney was very light, therefore we cut the renal capsule at the renal hilum after bluntly separating in the loose plane under the renal capsule. Then the renal pedicle arteries and veins were together clamped, ligated, and cut, and the ureter was exposed and cut. Finally, the transplanted kidney was removed completely. During the operation, we had paid great attention to avoid massive hemorrhage caused by avulsion of the renal pedicle and antibiotics had been prophylactically used to prevent infection. The patient received regular hemodialysis treatment after the operation.

**Figure 2:**
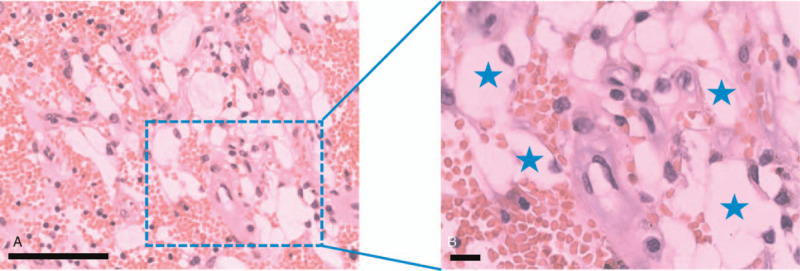
H&E staining of tissue sections shows clear cells in the renal transplant (shown by asterisks in B). Combined with the immunohistochemical staining, it supports locally acquired cystic disease-associated renal cell carcinoma. Scale bar = 50 μm.

Gross observation showed that the transplanted kidney had atrophic changes. The tumor is located in the cortex of the transplanted kidney, with a convex growth, about 2.5 × 1.5 cm in diameter, and an intact capsule. After longitudinal incision, it was found that the tumor was multilocular cystic with a thick wall and granular inner surface (Fig. 1B, C). Histopathological examination revealed locally acquired cystic disease-associated renal cell carcinoma with focal hemorrhage, degeneration, fibrosis, and necrosis (Fig. 2). The patient recovered well and was discharged 6 days after the operation. At the 12th month postoperation, no residual tumor or recurrence was found by imaging, and the creatinine level of the patient was about 3.5 mg/dL under hemodialysis treatment. The patient planned to have second kidney transplantation 6 months later, so we recommend that the immunosuppressive drug regimen be adjusted to mycophenolate mofetil because of the potential toxicity of cyclosporine A to the kidney.

## 3 Discussion

ACKD-RCC is a unique subtype of RCC found exclusively in patients with end-stage renal disease. It has unique pathological characteristics and is extremely rare in clinic. Tillou et al reported retrospective cohort studies in 32 renal transplantation centers that the incidence of renal graft tumor after transplantation is 0.19% (79 in 41,806 recipients). The incidence of non-functional renal graft tumor is as low as 0.032% (18 in 56,806 recipients).^[4,5]^ Most RCCs are diagnosed fortuitously, and their prevalence is underestimated.

The pathogenesis of ACRD-RCC so far has not been fully clarified. The existing theories include multifactorial reasons as immunosuppression, ESRD, oncogenic viruses, natural kidney evolution, dialysis, et al.^[6,7]^ First, several transplant and immunological factors might influence the risk of cancer after kidney transplantation, possibly owing to differences in rates of graft rejection and overall exposure to immunosuppression.^[6]^ Continuous application of immunosuppressive drugs is a risk factor that cannot be ignored. This is because immunosuppressive drugs inhibit or terminate the immune surveillance of the body's own immune system on tumor cell generation and replication, resulting in a significant increase in the incidence rate of cancer. Among these drugs, the nephrotoxicity of cyclosporine A is considered to directly promote the occurrence of kidney tumors.^[8]^ Second, the cancer risk after transplantation is also affected by the underlying cause of ESRD. Long term hemodialysis also promotes the formation of ACRD in patients with renal allograft dysfunction.^[6]^ ACRD can further cause local hemorrhage or malignant changes. Third, papillary adenomas and associated cysts have also been considered as putative precursors of this neoplasm.^[3,9]^ There are currently no guidelines on the management of de novo graft tumors in renal transplant patients.^[10]^ The basic principle is to preserve the function of the transplanted kidney as much as possible on the basis of complete resection of the primary tumor. The size and location of the tumor, the function of the graft and the general condition of the patient are the main factors affecting the choice of treatment.^[11]^ Graft nephrectomy is usually the first choice to improve the prognosis of the tumor, especially for non-functional graft kidney.^[∗]^ And the potential worse prognosis of tumors in immunosuppressed patients has also motivated urologists to perform graft nephrectomy as a first-line treatment. However, graft nephrectomy brings the patient back to hemodialysis, which is associated with a reduced life expectancy.^[8]^ Thus, when there is the only functioning kidney in patients with a renal tumor, it is also preferable to attempt nephron-sparing surgery, including partial nephrectomy, radiofrequency ablation, and cryoablation.^[12,13]^ In this case, we consider transplant nephrectomy surgery as an early treatment strategy for a non-functional kidney allograft with cystic degeneration to reduce the dosage of antirejection drugs, avoid the occurrence of transplanted kidney tumors, and provide the possibility for the patient to receive a second kidney transplant. Our clinical practice shows that ICAN has the following advantages. First, the safety of the operation is guaranteed. Due to scar hyperplasia and long-term host-to-graft reaction in the transplantation area, the tissue around the transplanted kidney appeared severe adhesion. Sharp separation or forced dissection may cause severe damage to surrounding vital organs and massive bleeding. Second, the margin of tumor tissue can be guaranteed to be negative. Because the tumor capsule is complete and thick, separating the tumor at the boundary outside the tumor capsule and inside the renal capsule can avoid the possibility of local tumor residue. Third, the graft was removed within the maximum range allowed by existing technical conditions, which reduced the possibility of producing host antigraft antibodies, reduced the dosage of antirejection drugs, and provided the opportunity for the second renal transplantation in the future. In conclusion, there is no unified international guideline for the screening, diagnosis, and treatment of ACRD-RCC. With the continuous optimization of renal replacement therapy, the life span of patients with end-stage renal disease is gradually extending. Therefore, ACRD patients on long-term hemodialysis need close review and reliable follow-up. Regular monitoring of non-functional grafts should be performed with at least an annual ultrasonography.

## Author contributions

**Conceptualization:** Yue Song, Jingjing Zheng, Shiying Guo, Lianhui Fan.

**Data curation:** Yue Song, Jingjing Zheng, Lianhui Fan.

**Formal analysis:** Yue Song, Jingjing Zheng, Lianhui Fan.

**Funding acquisition:** Yue Song, Jingjing Zheng.

**Investigation:** Yue Song.

**Methodology:** Yue Song, Jingjing Zheng, Shiying Guo.

**Resources:** Yue Song.

**Software:** Yue Song.

**Supervision:** Shiying Guo, Lianhui Fan.

**Visualization:** Yue Song.

**Writing – original draft:** Yue Song, Jingjing Zheng.

**Writing – review & editing:** Yue Song, Jingjing Zheng.

## References

[1] Hogg RJ. Acquired renal cystic disease in children prior to the start of dialysis. Pediatr Nephrol 1992;6:176–8. 1571216 10.1007/BF00866306

[2] Chung WY Nast CC Ettenger RB. Acquired cystic disease in chronically rejected renal transplants. J Am Soc Nephrol 1992;2:1298–301. 1627755 10.1681/ASN.V281298

[3] Tickoo SK dePeralta-Venturina MN Harik LR. Spectrum of epithelial neoplasms in end-stage renal disease: an experience from 66 tumor-bearing kidneys with emphasis on histologic patterns distinct from those in sporadic adult renal neoplasia. Am J Surg Pathol 2006;30:141–53. 16434887 10.1097/01.pas.0000185382.80844.b1

[4] Vaudreuil L Bessede T Boissier R. De novo renal carcinoma arising in non-functional kidney graft: a national retrospective study. Int Urol Nephrol 2020;52:1235–41. 32107673 10.1007/s11255-020-02422-0

[5] Tillou X Doerfler A Collon S. De novo kidney graft tumors: results from a multicentric retrospective national study. Am J Transplant 2012;12:3308–15. 22959020 10.1111/j.1600-6143.2012.04248.x

[6] Au E Wong G Chapman JR. Cancer in kidney transplant recipients. Nat Rev Nephrol 2018;14:508–20. 29802400 10.1038/s41581-018-0022-6

[7] Przybycin CG Harper HL Reynolds JP. Acquired cystic disease-associated renal cell carcinoma (ACD-RCC): a multiinstitutional study of 40 cases with clinical follow-up. Am J Surg Pathol 2018;42:1156–65. 29851703 10.1097/PAS.0000000000001091

[8] Rao PS Schaubel DE Jia X. Survival on dialysis post-kidney transplant failure: results from the Scientific Registry of Transplant Recipients. Am J Kidney Dis 2007;49:294–300. 17261432 10.1053/j.ajkd.2006.11.022

[9] Sun Y Argani P Tickoo SK. Acquired cystic disease-associated renal cell carcinoma (ACKD-RCC)-like cysts. Am J Surg Pathol 2018;42:1396–401. 30001236 10.1097/PAS.0000000000001124

[10] Scott MH Sells RA. Primary adenocarcinoma in a transplanted cadaveric kidney. Transplantation 1988;46:157–8. 3293278

[11] Sarantitis I Pararajasingam R Forgacs B. Transperitoneal enucleation of a kidney transplant allograft renal cell carcinoma. Exp Clin Transplant 2018;16:614–6. 27855588 10.6002/ect.2016.0037

[12] Chambade D Meria P Tariel E. Nephron sparing surgery is a feasible and efficient treatment of T1a renal cell carcinoma in kidney transplant: a prospective series from a single center. J Urol 2008;180:2106–9. 18804233 10.1016/j.juro.2008.07.055

[13] Tsuboi I Araki M Fujiwara H. Contrast-enhanced computed tomography-guided percutaneous cryoablation of renal cell carcinoma in a renal allograft: first case in Asia. Acta Med Okayama 2019;73:269–72. 31235976 10.18926/AMO/56871

